# Evaluating the Negative Appendicectomy Rate: A Retrospective Observational Cohort Study at a UK Teaching Hospital Trust

**DOI:** 10.7759/cureus.97359

**Published:** 2025-11-20

**Authors:** Jin Ren Lau, Ker Yin Tee, Karim Heiba, Ahsan Ali Shahbaz, Mohamed Elhennawi, Muhammad A Butt

**Affiliations:** 1 General Surgery, East Lancashire Hospitals NHS Trust, Blackburn, GBR; 2 Internal Medicine, East Lancashire Hospitals NHS Trust, Blackburn, GBR; 3 Emergency Medicine, East Lancashire Hospitals NHS Trust, Blackburn, GBR

**Keywords:** acute appendicitis, diagnostic accuracy, laparoscopic appendicectomy, nar, negative appendicectomy rate, united kingdom

## Abstract

Introduction

The negative appendicectomy rate (NAR) serves as a key quality indicator in surgical practice, reflecting diagnostic accuracy and decision-making in suspected acute appendicitis. Despite improvements in imaging and clinical scoring systems, NAR in the United Kingdom remains higher than international averages. This study aimed to determine the NAR at East Lancashire Hospitals NHS Trust (ELHT), compare it with published benchmarks, and evaluate factors influencing diagnostic accuracy and clinical decision-making.

Methods

A retrospective observational cohort study was conducted at ELHT, encompassing all emergency appendicectomies performed between June 2023 and June 2024. Data were retrieved from electronic patient records and included demographics, clinical presentation, laboratory findings, imaging modality, and histopathology. Negative appendicectomy was defined histologically as the absence of inflammatory changes in the appendix. Subgroup analyses were performed by age, gender, and imaging use. Postoperative complications in negative appendicectomy cases were graded according to the Clavien-Dindo classification. A clinician survey assessed the utilisation of diagnostic scoring systems. Statistical analysis was performed using IBM SPSS Statistics version 31, with *p* < 0.05 considered significant.

Results

Of the 320 patients included, negative appendicectomy occurred in 59 cases (18.4%), with the NAR consistent with national data but higher than the international pooled estimate of 13%. Among 214 adults, the NAR was 15.9% (34); while among 106 children, the NAR was 23.6% (25) (p=0.0947). Female patients had a significantly higher NAR of 25.6% (33) compared with males at 13.6% (26) (p=0.0068). Preoperative imaging was performed in 176 cases (55.0%), with 50.8% (30) of negative appendicectomies occurring without imaging. Postoperative complications were observed in 6 negative cases (10.2%), all within Clavien-Dindo grade I-II. Thirty clinician survey responses revealed limited use of validated scoring systems in adult appendicitis, with 20 clinicians (66.7%) relying primarily on clinical intuition and experience.

Conclusion

The institutional NAR of 18.4% aligns with national figures but remains above international benchmarks. Higher rates among paediatric and female patients highlight persistent diagnostic challenges. Standardising diagnostic pathways through consistent use of validated clinical scoring tools such as the AIR and AAS scores, coupled with appropriate imaging, may enhance diagnostic accuracy, reduce unnecessary surgery, and improve patient outcomes.

## Introduction

Acute appendicitis (AA) is one of the most common causes of lower abdominal pain leading to presentation at emergency departments and remains the most frequent abdominal surgical emergency worldwide [1]. In the United Kingdom, the annual incidence is approximately 81 cases per 100,000 population, with around 50,000 appendicectomies performed each year [1,2]. AA demonstrates a peak incidence in the first to third decades of life, while mortality risk is highest at the extremes of age [1,3].

Since its introduction in the nineteenth century, appendicectomy has remained the mainstay of treatment for AA [3]. Laparoscopic appendicectomy is now favoured over the open approach due to its advantages in reducing surgical site infections, postoperative pain, and hospital length of stay, while improving postoperative recovery and quality of life [3,4]. Although generally safe and providing definitive resolution, appendicectomy carries morbidity rates ranging from 2% to 23%, including postoperative ileus, wound infection, intra-abdominal abscess, incisional hernia, and adhesions, and is associated with the occurrence of negative appendicectomy [3,5].

In recent years, non-operative management with antibiotics has gained increasing interest as a primary treatment strategy for uncomplicated appendicitis. This approach is associated with lower rates of immediate postoperative complications, with a reported 39% relative risk reduction compared to surgery [6,7]. However, despite these short-term benefits, recent meta-analyses consistently demonstrate that conservative management is associated with higher treatment failure rates [6-8], with approximately one in five patients requiring readmission or subsequent appendicectomy within one year [6]. Although evidence supports the feasibility of antibiotic therapy in selected cases, no consensus exists regarding its routine use as primary treatment for uncomplicated AA, and substantial variability persists in clinical practice [3].

Diagnosis of AA remains challenging and typically relies on a combination of clinical evaluation, biochemical testing, and radiological imaging [3]. This difficulty is amplified in children, where atypical presentations occur in nearly half of all cases and reliable history-taking may be limited [9]. To aid diagnostic decision-making and reduce uncertainty, several risk-prediction models have been developed, including the Alvarado score, Appendicitis Inflammatory Response (AIR) score, modified Alvarado score and Adult Appendicitis Score (AAS), with the Paediatric Appendicitis Score (PAS) tailored for children [10-14]. While these scoring systems are not diagnostic tools in isolation, they are most effective when integrated into structured diagnostic pathways alongside imaging [3].

Over recent decades, advances in imaging and the implementation of structured diagnostic algorithms have significantly reduced the negative appendicectomy rate (NAR). A meta-analysis demonstrated a decline in NAR from 21.5% in the pre-CT era to approximately 10% following the widespread adoption of computed tomography [15]. In the United Kingdom, however, the rate remains comparatively high at around 20.6% as reported by the National Surgical Research Collaborative multicentre study [16]. Internationally, the mean NAR is estimated at approximately 13% (95% CI 12-14%) [17], highlighting the continued need to enhance diagnostic accuracy and standardise management pathways for appendicitis across UK clinical settings.

Despite its clinical relevance, there remains no universally accepted definition of a negative appendicectomy. Traditionally, it describes the removal of an appendix that appears macroscopically normal intraoperatively and/or lacks histological evidence of acute inflammation. A stricter definition relies solely on histopathological findings, specifically the absence of mucosal or wall infiltration by polymorphonuclear leukocytes, lymphocytes, or plasma cells [18]. An international survey of surgeons from 39 countries demonstrated substantial heterogeneity in definitions, with most identifying a histologically normal appendix as the defining criterion [19]. This variability underscores the need for standardised diagnostic and pathological definitions to ensure consistency in reporting and comparison of NAR across studies and institutions.

NAR is widely recognised as a key quality indicator in surgical practice, reflecting the accuracy of diagnostic pathways and clinical decision-making. A high NAR indicates unnecessary surgery with associated morbidity and cost, whereas an excessively low NAR may suggest diagnostic delay and increased risk of perforation. Most surgeons consider a rate below 10% to be acceptable [19]. In the UK context, where NAR remains comparatively high, national and local initiatives continue to focus on optimising diagnostic accuracy through standardised pathways, improved imaging utilisation, and incorporation of validated scoring systems. Monitoring NAR at the institutional level provides a meaningful indicator of diagnostic performance, surgical quality, and overall healthcare efficiency.

## Materials and methods

Study population

This retrospective single-centre observational study was conducted at East Lancashire Hospitals NHS Trust (ELHT), which includes Royal Blackburn Teaching Hospital (RBH) and Burnley General Teaching Hospital (BGH). Both hospitals provide acute secondary healthcare services for the East Lancashire region. Royal Blackburn Teaching Hospital serves as the trust’s primary site for emergency surgical operations, while the Urgent Treatment Centre at Burnley General Teaching Hospital functions as an acute assessment and transfer unit for patients requiring urgent operative intervention.

**Figure 1:**
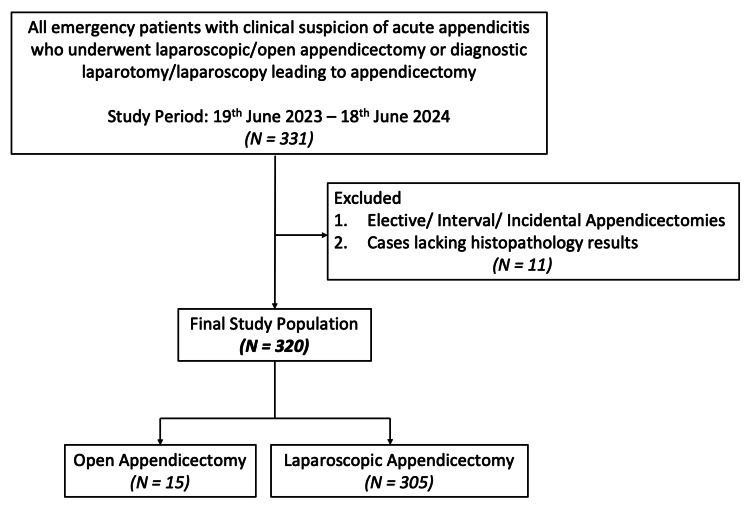
Cohort flowchart

The study included all emergency patients admitted with a clinical suspicion of acute appendicitis who underwent laparoscopic or open appendicectomy, or diagnostic laparoscopy or laparotomy leading to appendicectomy, over a one-year period between 19th June 2023 (coinciding with the introduction of the electronic patient record system) and 18th June 2024. Patients were identified retrospectively from the emergency theatre electronic operating list. Inclusion criteria included all patients of any age and gender who underwent emergency appendicectomy within the defined study period. Adults were classified as patients aged 18 years and above, and paediatric patients as those under 18 years of age. A total of 331 patients were initially identified. Exclusion criteria included elective, interval, or incidental appendicectomies, as well as cases lacking histopathology results (N=11). Following exclusions, a final sample of 320 patients was included in the analysis, of which 305 underwent laparoscopic appendicectomy and 15 underwent open appendicectomy. The cohort flowchart is illustrated in Figure 1.

Data collection

Data were extracted from electronic patient records using the Cerner Millennium system and the Sunquest ICE laboratory database. Variables collected included demographic data (age and gender), clinical presentation, laboratory findings and imaging results from ultrasound and computed tomography (CT) scans. Operative details and postoperative complications were reviewed from the operation and discharge documentation. Histopathology reports were reviewed for diagnostic confirmation. A negative appendicectomy was defined according to standard histological criteria, as the absence of inflammatory changes or other significant pathological abnormalities in the appendicular specimen, as described by Noureldin et al. [18]. Subgroup analyses were performed according to age, gender, and imaging modality to evaluate diagnostic variation. Postoperative complications were graded according to the Clavien-Dindo classification, which categorises complications from Grade I (minor deviation from normal recovery not requiring treatment), Grade II (requiring pharmacological treatment), Grade III (requiring surgical, endoscopic, or radiological intervention), Grade IV (life-threatening complications requiring intensive care), and Grade V (death) [20].

An additional local clinician survey was conducted, yielding 30 responses. The survey targeted members of the surgical team, including senior house officers (SHOs), registrars, and consultants, to assess familiarity with and use of clinical scoring systems in the diagnostic workup and risk stratification of suspected appendicitis. The findings provided insight into current local practice patterns and the reliance on clinical judgement versus structured scoring methods.

Statistical analysis

Data were compiled and analysed using Microsoft Excel 2024 and IBM SPSS Statistics version 31. Descriptive statistics were used to summarise patient demographics and clinical parameters. Continuous variables were expressed as mean ± standard deviation (SD) or median with interquartile ranges (IQR), while categorical data were presented as frequencies and percentages. The negative appendicectomy rate (NAR) was calculated as the proportion of histologically normal appendices among all appendicectomies performed. Subgroup comparisons by gender, age group, and imaging modality were conducted using the Pearson Chi-square test or Fisher’s exact test for categorical variables, with p<0.05 considered statistically significant. The results were benchmarked against the systematic review and meta-analysis by Henriksen et al., which reported a pooled NAR of 12-14% following laparoscopic appendicectomy, and further compared with findings from recent cohort studies to contextualise institutional performance [17].

This study was registered as a clinical audit and approved by the ELHT Clinical Audit & Effectiveness Team. Formal ethical approval was not required, in accordance with NHS Health Research Authority guidance, as the project was defined as a clinical audit.

## Results

Patient cohort

A total of 320 patients were included in this study, of which 59 cases (18.4%) had negative appendicectomy. Among those who underwent laparoscopic appendicectomy (N=305; 95.3%), the NAR was 18.4% (n=56); while among open appendicectomy cases (N=15; 4.7%), the NAR was 20.0% (n=3), showing no significant difference between surgical approaches (p=0.745). The distribution of surgical approaches and corresponding NARs is summarised in Table 1.

**Table 1:** Negative appendicectomy rate by surgical approach n, negative appendicectomy cases; N, total appendicectomy cases; NAR, negative appendicectomy rate

Surgical approach	n	N	NAR (% = n/N x 100)	p-value
Laparoscopic cases	56	305	18.4	0.745
Open cases	3	15	20.0	
Overall	59	320	18.4	-

Age distribution

Patient age ranged from 6 to 88 years (median 27 years). There were 214 adults (66.9%) and 106 children (33.1%). Among adults, 34 (15.9%) had negative histology, compared with 25 (23.6%) among children (p=0.0947). The median age of patients with negative histology was 22 years, while the median age of those with positive histology was 28 years.

Gender

The cohort comprised 191 males (59.7%) and 129 females (40.3%). Of these, 26 males (13.6%) and 33 females (25.6%) had negative histology (p=0.0068).

Preoperative imaging

Preoperative imaging was performed in 176 (55.0%) cases overall. Among patients with negative appendicectomy (n=59, 18.4%), 30 (50.8%) underwent surgery without any preoperative imaging, 19 (32.3%) had ultrasound (USS), and 10 (16.9%) underwent computed tomography (CT). The breakdown of preoperative imaging is demonstrated in Table 2. 

**Table 2:** Preoperative imaging in negative appendicectomy cases (n=59) n, negative appendicectomy cases

Imaging	n	%
No Imaging	30	50.8
Ultrasound	19	32.3
CT	10	16.9

Post-operative complications in negative appendicectomy cases

Among the negative appendicectomy cases, 6 patients (10.2%) developed postoperative complications within 30 days of surgery, as demonstrated in Table 3. All complications were graded as Clavien-Dindo I-II, with no Grade III-V events or reoperations reported. These findings indicate a low 30-day morbidity rate following negative appendicectomy, with all cases managed conservatively and no readmissions beyond minor interventions.

**Table 3:** Post-operative complications in negative appendicectomy cases (n=59) based on Clavien-Dindo Classification n, negative appendicectomy cases; Clavien-Dindo Classification [20]

Grade	Complications in negative appendicectomy cases	n	%
0	No complication	53	89.8
I	Wound infection opened at bedside	2	3.4
	Antiemetics for vomiting	1	1.7
	Analgesia for abdominal pain	1	1.7
II	Antibiotics for wound infection	1	1.7
	PPI for GERD	1	1.7
III - V	-	0	0

Survey on diagnostic scoring systems

A survey was conducted among 54 surgical doctors, including senior house officers (SHOs), trainees, registrars, and consultants, with a total of 30 responses (55.6% response rate). Respondents were allowed to select more than one option to reflect their actual diagnostic practice across both adult and paediatric appendicitis.

Adult Appendicitis

**Figure 2:**
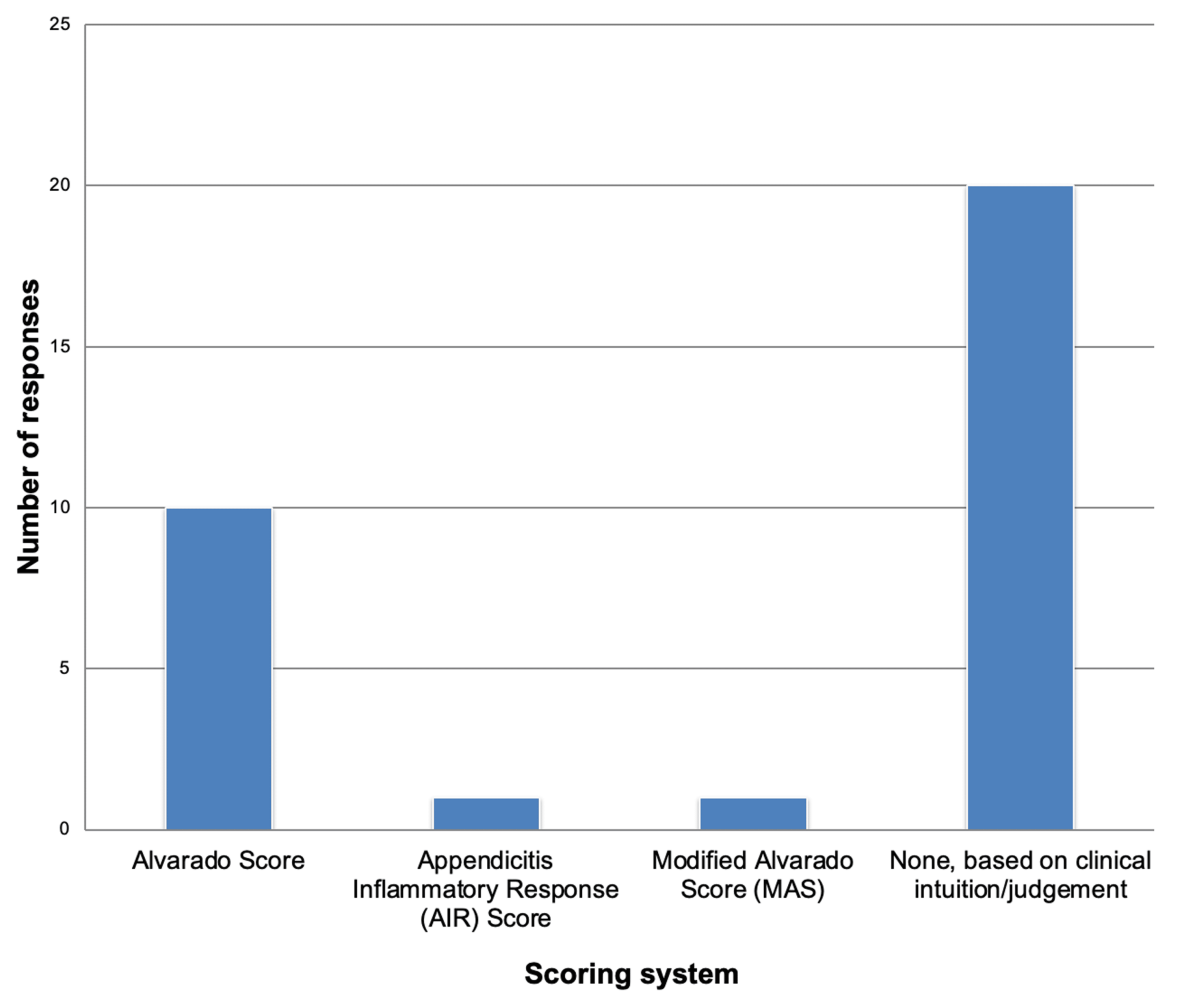
Frequency of diagnostic scoring systems used among respondents (Adult Appendicitis) Alvarado Score [10], Appendicitis Inflammatory Response (AIR) Score [11], Modified Alvarado Score (MAS) [12]

For adult appendicitis, the majority of respondents (n=20; 66.7%) indicated that they relied primarily on clinical intuition and judgment based on experience. The Alvarado Score was the most frequently used structured tool, applied by 10 respondents (n=10; 33.3%), while a small number reported use of the Modified Alvarado Score (n=1; 3.3%) and the Appendicitis Inflammatory Response (AIR) Score (n=1; 3.3%). These are demonstrated in Figure 2.

Paediatric Appendicitis

**Figure 3:**
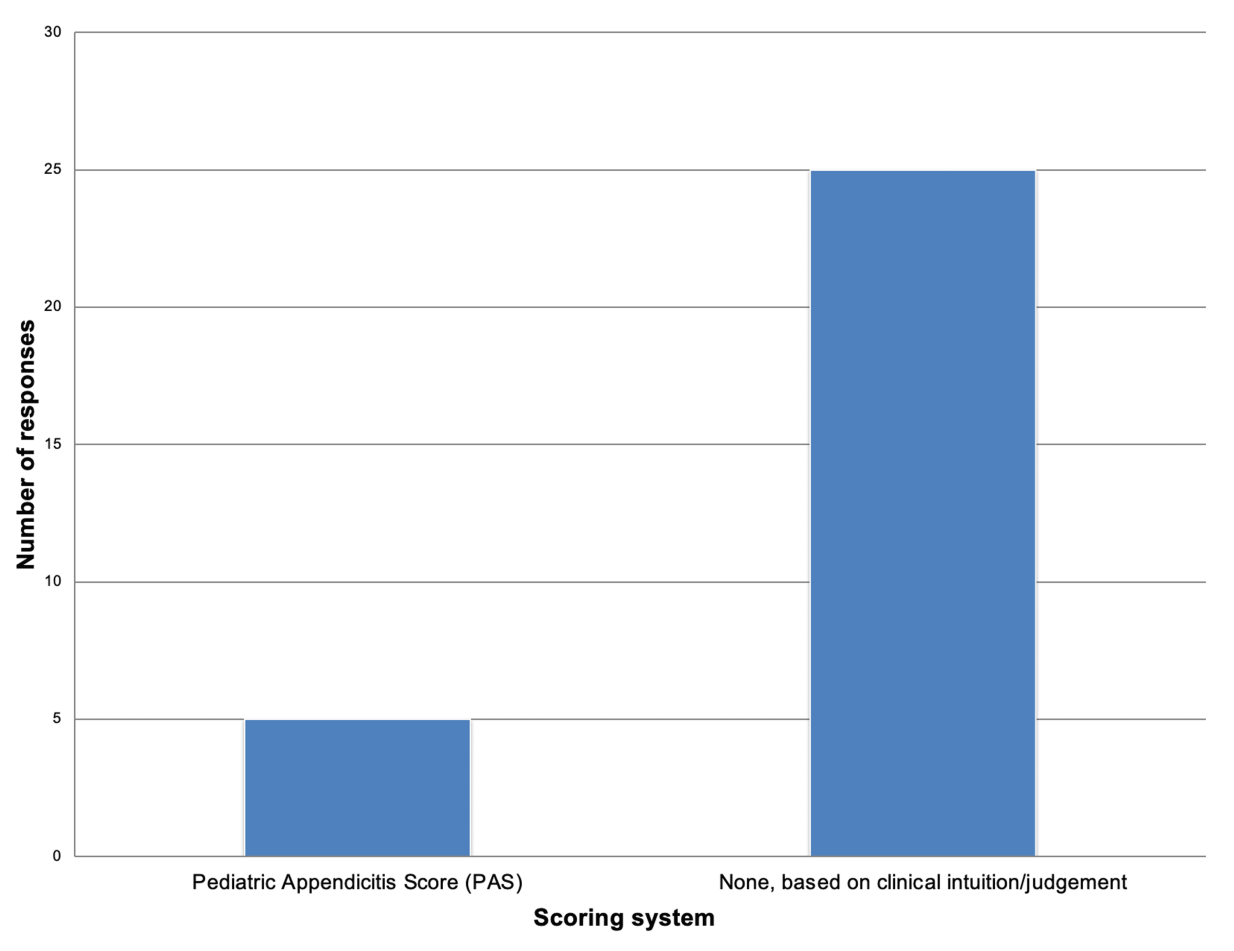
Frequency of diagnostic scoring systems used among respondents (Paediatric Appendicitis) Pediatric appendicitis score (PAS) [14]

In paediatric cases, a large majority of respondents (n=25; 83.33%) reported diagnosing primarily based on clinical intuition and judgment, while only 5 respondents (16.7%) indicated using the Paediatric Appendicitis Score (PAS). These are demonstrated in Figure 3. Summary of main findings as demonstrated in Table 4.

**Table 4:** Summary of main findings n, negative appendicectomy cases; N, total appendicectomy cases; NAR, negative appendicectomy rate

Variable	n	N	NAR (% = n/N x 100)	p-value
Overall	59	320	18.4	-
Laparoscopic cases	56	305	18.4	0.745
Open cases	3	15	20.0	
Adults (≥ 18 y)	34	214	15.9	0.0947
Children (< 18 y)	25	106	23.6	
Male	26	191	13.6	0.0068
Female	33	129	25.6	
Imaging (USS/ CT)	29	176	16.5	0.3175
No imaging	30	144	20.8	

## Discussion

Acute appendicitis remains the most common abdominal surgical emergency [1]. However, diagnostic challenges persist and can lead to unnecessary operations, postoperative complications, and increased healthcare costs. The negative appendicectomy rate (NAR) acts as a key quality indicator of diagnostic accuracy and clinical decision-making. This study evaluates the NAR at East Lancashire Hospitals NHS Trust (ELHT) against published benchmarks and examines contributing factors, including patient age, gender, imaging utilisation, and the use of clinical scoring systems. The discussion interprets these findings in the context of current literature, identifies areas for improvement in diagnostic pathways, and outlines strategies to reduce unnecessary appendicectomies while maintaining patient safety.

Overall negative appendicectomy rate

Our study reports an overall negative appendicectomy rate (NAR) of 18.4% (n=59), which is slightly higher than the 13% (95% CI 12-14%) pooled estimate reported by Henrikson et al. in their systematic review and meta-analysis [17]. Nevertheless, this rate remains consistent with previously published UK data, including the multicentre study by the National Surgical Research Collaborative, which reported a negative appendicectomy rate of 20.6% and highlighted comparable variability across NHS trusts [15].

Surgical approach

In our study, the majority of patients underwent laparoscopic appendicectomy (N=305, 95.3%), reflecting the global shift toward minimally invasive techniques. This approach is increasingly preferred because of its association with lower postoperative complication rates, reduced pain, shorter hospital stay, faster recovery, improved quality of life, and potentially lower healthcare costs when sufficient expertise and resources are available [3,4,21]. These findings align with the World Society of Emergency Surgery (WSES) 2020 Guidelines, which recommend laparoscopic appendicectomy as the preferred technique for both uncomplicated and complicated appendicitis where feasible [3].

The NAR in our cohort was 18.4% (n=56) for laparoscopic cases and 20.0% (n=3) for open cases. Statistical comparison using Fisher’s exact test demonstrated no significant difference between the two approaches (p=0.745). This observation supports the notion that diagnostic accuracy and decision-making quality are similar between the two methods. Our results are consistent with Augustin et al., who found no significant difference in NAR between laparoscopic and open appendicectomy [22]. However, Akbar et al. found a higher NAR associated with laparoscopic surgery, suggesting that the minimally invasive nature of laparoscopy may prompt earlier exploration in diagnostically uncertain cases, particularly among women, which leads to the removal of a greater number of normal appendices [21]. Such variability between studies likely reflects differences in patient selection, operative thresholds, and the availability of imaging or diagnostic adjuncts. Additionally, in accordance with WSES 2020 guidelines and the recommendations of both the European Association of Endoscopic Surgery (EAES) 2016 and the Society of American Gastrointestinal and Endoscopic Surgeons (SAGES) 2010 guidelines, intraoperative macroscopic assessment of early appendicitis is often unreliable and varies between surgeons [3]. Therefore, removal of a macroscopically normal appendix is considered appropriate when no alternative intra-abdominal pathology is identified in a symptomatic patient.

Age distribution

In our cohort, patients aged 18 years and above accounted for 214 (66.9%) of all cases, with a negative appendicectomy rate of 15.9% (n=34). In comparison, paediatric patients (<18 years) comprised 106 (33.1%) of the total, with a higher NAR of 23.6% (n=25), exceeding the 15.9% benchmark reported by the RIFT study for paediatric populations in the UK and Ireland [23].

Although more adults underwent appendicectomy in our cohort, the higher paediatric NAR likely reflects the complex diagnostic challenges inherent to this group. Children frequently present with atypical or non-classical symptoms, where studies suggest that up to 50% of paediatric appendicitis cases exhibit atypical features, complicating clinical assessment and increasing the risk of misdiagnosis [9,24]. Diagnostic uncertainty in children is further compounded by communication barriers and the difficulty of physical examination in younger patients, which limits the reliability of both history and clinical findings [9]. The risk of misdiagnosis is particularly elevated among female children, who are more frequently subjected to unnecessary surgery due to overlapping presentations with gynaecological or functional abdominal pain syndromes [24,25].

Gender distribution

In our cohort, 191 (59.7%) were males and 129 (40.3%) were females. The NAR was significantly higher in females (25.6%, n=33) compared with males (13.6%, n=26), consistent with previously published studies [21,26]. Although appendicitis is more common in males, females, and particularly those of reproductive age, are at higher risk of negative appendicectomy due to the diagnostic overlap with gynaecological conditions such as ovarian cysts, pelvic inflammatory disease, and endometriosis. One study also reported that women of childbearing age may have an NAR as high as 43%, compared with 35% in the general population [27]. Imaging choice likely contributes to this difference. In our cohort, CT was used more frequently in male patients, whereas ultrasound was the predominant imaging modality in females, primarily to avoid radiation exposure in reproductive-age women. This pattern mirrors findings in the wider literature, where the lower sensitivity and operator dependence of ultrasound, compared with CT, can increase diagnostic uncertainty and NAR in females [27]. 

Imaging use

Preoperative imaging was performed in 176 (55.0%) cases across the entire cohort. Among the 59 (18.4%) patients who underwent negative appendicectomy, 30 (50.8%) proceeded to surgery without any preoperative imaging, 19 (32.3%) had an ultrasound, and 10 (16.9%) underwent a CT scan. Although ultrasound remains a valuable first-line, non-invasive, and radiation-free modality, especially in younger and female patients, its diagnostic performance is limited by operator dependence, patient body habitus, and interference from bowel gas [18]. In contrast, CT imaging offers superior diagnostic accuracy and is already well integrated into diagnostic pathways in our cohort. 

Enhanced adherence to evidence-based diagnostic pathways, such as those recommended by the WSES, could further optimise clinical decision-making [3]. Broader yet judicious use of CT in uncertain cases, alongside ultrasound in lower-risk or radiation-sensitive groups, and the integration of validated clinical scoring systems (e.g., AIR or AAS scores), may reduce diagnostic uncertainty. Ultimately, aligning operative decision-making with both clinical and imaging evidence could substantially decrease the negative appendicectomy rate while maintaining patient safety.

Postoperative complications

Among the 59 (18.4%) patients with negative appendicectomy, 6 (10.2%) experienced postoperative complications within 30 days, all of which were minor (Clavien-Dindo grade I-II) and did not require re-intervention. This is consistent with the 5-15% postoperative morbidity rate reported in the literature [26,28]. Although minor, these complications, such as wound infections or prolonged postoperative pain, can delay recovery, extend hospital stay, and increase healthcare costs. Improving preoperative diagnostic accuracy through standardised clinical scoring and appropriate imaging is essential to reducing unnecessary operations and their associated complications. Our study is limited by its retrospective design and short-term (30-day) follow-up, which may underestimate long-term morbidity such as adhesional complications or chronic pain. Furthermore, minor complications presenting after discharge may not have been captured in hospital records, potentially leading to underreporting. Prospective studies with longer follow-up are needed to provide a more comprehensive assessment of the postoperative burden associated with negative appendicectomy.

Use of clinical scoring systems

Our clinician survey revealed that 66.7%, which is 20 of 30 respondents, including most registrars and consultants, do not routinely employ clinical scoring systems when assessing patients with suspected adult appendicitis, instead relying primarily on clinical acumen and experience. Among those who did utilise scoring, the Alvarado score was most frequently applied in adults, while only one clinician reported using the Appendicitis Inflammatory Response (AIR) score.

Although the Alvarado score is familiar and widely used, it omits key inflammatory markers such as C-reactive protein (CRP). The AIR score compensates for this by including both CRP and white cell count (WCC) alongside graded clinical findings, providing superior diagnostic performance, particularly in intermediate-risk patients and those with complex presentations such as women and children [11]. Studies by Kularatna et al. and subsequent validation research have shown the AIR score to achieve sensitivities of 92-98%, specificity up to 88%, and a higher area under the ROC curve than the Alvarado score [29]. Consequently, international bodies such as the World Society of Emergency Surgery (WSES) recommend preferential use of the AIR or AAS scores for clinical decision-making [3].

In the paediatric population, the Paediatric Appendicitis Score (PAS) was used by only 16.7% of clinicians (five clinicians). Although both the Alvarado and PAS scores are recognised by WSES as useful for excluding appendicitis in children [3], newer evidence suggests that the AIR score may offer greater diagnostic value in this group as well. Macco et al. demonstrated that the AIR score outperformed both the Alvarado and PAS scores in predicting appendicitis among paediatric patients [30].

Given this evidence, there is clear potential to standardise the use of validated scoring systems such as the AIR and AAS within our trust. Incorporating these tools into the initial assessment of suspected appendicitis could enhance diagnostic accuracy, improve imaging utilisation, and reduce unnecessary surgery. Standardising practice in line with international recommendations would ultimately enhance patient safety, optimise resource use, and contribute to lowering the negative appendicectomy rate.

Limitations

Our study represents a single-centre retrospective analysis, which may limit external validity and the generalisability of findings to other institutions or populations. The study relied on the accuracy and completeness of existing electronic patient records, introducing potential for documentation bias. In addition, the absence of histological confirmation in some cases limited inclusion in the final analysis and may have led to selection bias. The study was also limited to comparisons based on standard criteria for negative appendicectomy, as the degree of inflammatory change could not be uniformly classified. The lack of detailed or consistent microbiological and histopathological reporting prevented the application of stricter definitions for negative appendicectomy [18].

This retrospective design of our study also precludes control over patient selection, diagnostic decision-making, and intraoperative management. The decision to perform appendicectomy was largely based on the clinical judgment of the operating surgeon rather than a standardised pre-defined protocol, introducing variability in diagnostic thresholds and operative indications that is inherent to retrospective studies.

Thirdly, it also remains routine surgical practice to remove an appendix that appears macroscopically normal when no other intra-abdominal pathology is identified in a symptomatic patient [3]. Although this approach aims to prevent missed cases of early or atypical appendicitis, it may contribute to a higher negative appendicectomy rate.

Future studies should aim to overcome these limitations through prospective, standardised, and multicentre research designs that better reflect contemporary practice. A prospective cohort study incorporating structured data collection from the time of presentation to postoperative review would improve data reliability and allow a more comprehensive assessment of clinical, laboratory, and intraoperative factors associated with negative appendicectomy.

The findings of this study also emphasise the importance of continued audit and feedback in appendicitis management. Periodic review of NAR trends, combined with multidisciplinary case discussions, can identify modifiable factors and promote adherence to best practice. Ultimately, efforts should focus on improving diagnostic accuracy, minimising unnecessary surgery, and maintaining patient safety.

## Conclusions

Our study demonstrates an overall negative appendicectomy rate of 18.4%, comparable to national figures and reflective of ongoing diagnostic challenges, particularly among paediatric and female patients. Variability in imaging utilisation and limited adoption of validated clinical scoring systems could contribute to potentially avoidable surgeries. Standardising diagnostic pathways through consistent use of evidence-based scoring tools such as the AIR and AAS scores, coupled with imaging use, may enhance diagnostic accuracy, reduce unnecessary operations, and improve overall surgical outcomes.
